# Antibody against TDP-43 phosphorylated at serine 375 suggests conformational differences of TDP-43 aggregates among FTLD–TDP subtypes

**DOI:** 10.1007/s00401-020-02207-w

**Published:** 2020-08-10

**Authors:** Manuela Neumann, Petra Frick, Francesca Paron, Jonas Kosten, Emanuele Buratti, Ian R. Mackenzie

**Affiliations:** 1 Molecular Neuropathology of Neurodegenerative Diseases, German Center for Neurodegenerative Diseases (DZNE) Tübingen, Otfried-Müllerstr. 23, 72072 Tübingen, Germany; 2 Department of Neuropathology, University Hospital of Tübingen, Tübingen, Germany; 3 Department of Molecular Pathology, International Centre for Genetic Engineering and Biotechnology (ICGEB), Trieste, Italy; 4 Department of Pathology, University of British Columbia and Vancouver General Hospital, Vancouver, Canada

**Keywords:** TDP-43, Frontotemporal dementia, Frontotemporal lobar degeneration, ALS, Phosphorylation-specific antibody, TDP-43 strains, Conformation-specific

## Abstract

Aggregation of hyperphosphorylated TDP-43 is the hallmark pathological feature of the most common molecular form of frontotemporal lobar degeneration (FTLD–TDP) and in the vast majority of cases with amyotrophic lateral sclerosis (ALS–TDP). However, most of the specific phosphorylation sites remain to be determined, and their relevance regarding pathogenicity and clinical and pathological phenotypic diversity in FTLD–TDP and ALS–TDP remains to be identified. Here, we generated a novel antibody raised against TDP-43 phosphorylated at serine 375 (pTDP-43^S375^) located in the low-complexity domain, and used it to investigate the presence of S375 phosphorylation in a series (*n* = 44) of FTLD–TDP and ALS–TDP cases. Immunoblot analysis demonstrated phosphorylation of S375 to be a consistent feature of pathological TDP-43 species, including full-length and C-terminal fragments, in all FTLD–TDP subtypes examined (A–C) and in ALS–TDP. Of particular interest, however, detailed immunohistochemical analysis showed striking differences in the immunoreactivity profile of inclusions with the pTDP-43^S375^ antiserum among pathological subtypes. TDP-43 pathology of ALS–TDP, FTLD–TDP type B (including cases with the *C9orf72* mutation), and FTLD–TDP type C all showed strong pTDP-43^S375^ immunoreactivity that was similar in amount and morphology to that seen with an antibody against TDP-43 phosphorylated at S409/410 used as the gold standard. In stark contrast, TDP-43 pathology in sporadic and genetic forms of FTLD–TDP type A (including cases with *GRN* and *C9orf72* mutations) was found to be almost completely negative by pTDP-43^S375^ immunohistochemistry. These data suggest a subtype-specific, conformation-dependent binding of pTDP-43^S375^ antiserum to TDP-43 aggregates, consistent with the idea of distinct structural TDP-43 conformers (i.e., TDP-43 strains) as the molecular basis for the phenotypic diversity in TDP-43 proteinopathies.

**Electronic supplementary material:**

The online version of this article (10.1007/s00401-020-02207-w) contains supplementary material, which is available to authorized users.

## Introduction

Abnormal neuronal and glial inclusions composed of the transactive response DNA/RNA-binding protein M_r_ 43 kD (TDP-43) are the hallmark pathological lesions in two devastating neurodegenerative diseases subsumed as primary TDP-43 proteinopathies, frontotemporal lobar degeneration with TDP-43 pathology (FTLD–TDP), and in the vast majority of cases of amyotrophic lateral sclerosis (ALS–TDP) [1, 17]. FTLD–TDP is the most common molecular subtype in patients with frontotemporal dementia (FTD), a clinical syndrome characterized by progressive changes in behavior, personality, and/or language with the major clinical subtypes including the behavioral variant of frontotemporal dementia (bvFTD) and two forms of primary progressive aphasia (PPA); the non-fluent/agrammatic (nfvPPA) and semantic variants (svPPA) [30]. FTLD–TDP includes sporadic and genetic forms with mutations in *GRN*, *C9orf72, TBK1,* and *VCP* as the most common gene defects [24]. ALS–TDP is the most common form of motor neuron disease in which a predominant loss of motor neurons from the brain and spinal cord leads to fatal paralysis and death. Up to 10% of ALS cases are familial with *C9orf72* repeat expansion being the most common gene defect.

Despite the common feature of abnormal TDP-43 accumulation, a significant pathological heterogeneity is observed among TDP-43 proteinopathies with respect to the pattern of anatomical distribution and morphology of inclusions, allowing recognition of up to five distinct pathological subtypes of FTLD–TDP [12–14, 16]. Since their initial recognition in 2006 [15, 25], the concept and relevance of FTLD–TDP subtypes has further developed and gained wide acceptance by demonstrating that each of the different pathological subtypes is associated with relatively specific clinical and genetic correlations.

The molecular basis explaining the clinical and pathological phenotypic variability of TDP-43 proteinopathies is so far not understood. In analogy to prion diseases and other neurodegenerative diseases such as tau- and alpha-synucleinopathies, a popular hypothesis, supported by an increasing body of evidence, is that the heterogeneity is a reflection of biochemical differences in pathological TDP-43 species that propagate in a prion-like manner with different conformers being associated with distinct seeding/toxicity activities (i.e., TDP-43 strains) [5, 10, 11, 23, 29]. Biochemically, it is well established that aggregated TDP-43 becomes poorly detergent soluble and subject to a variety of disease associated post-translational modifications (PTM) including N-terminal truncation, phosphorylation, ubiquitination, acetylation, cysteine oxidation, and sumoylation [2]. However, the molecular properties of TDP-43 aggregates including specific sites of PTMs and their potential differences among distinct TDP-43 proteinopathies are not yet fully characterized.

Since its initial discovery as the disease protein in FTLD–TDP and ALS–TDP, aberrant phosphorylation of TDP-43 has been recognized as one major PTM of pathological TDP-43 [1, 17]. The fact that the majority of FTD/ALS-causing pathogenic mutations in the TDP-43 gene (*TARDBP*) either introduce or disrupt potential serine/threonine phosphorylation sites or introduce phosphomimic residues (glutamate/aspartate) [2] strongly suggests that alterations in the phosphorylation status of TDP-43 play a key role in TDP-43 pathogenesis and might contribute to biochemical heterogeneity of TDP-43 aggregates.

TDP-43 has 41 serine, 15 threonine, and 8 tyrosine residues that might act as potential phosphorylation sites. Mass spectrometry analyses of recombinant TDP-43 in vitro phosphorylated with casein kinase 1 and of aggregated TDP-43 isolated from ALS–TDP have revealed several phosphorylated residues [4, 8, 9]. However, so far, only five phosphorylation sites (serine 379, 403, 404, 409, 410) at the C-terminus of TDP-43 have been validated and studied in pathological TDP-43 inclusions in human post-mortem tissue using phosphorylation site-specific antibodies [4, 19]. Phosphorylation at serine 409/410 is by far the most thoroughly analyzed PTM, and is a highly consistent and specific feature of aggregated TDP-43 in all types of pathological TDP-43 inclusions in sporadic and familial ALS/FTLD–TDP subtypes [4, 19]. As a result, these pTDP-43^S409/410^ antibodies have facilitated the detection of TDP-43 pathology and are considered to be the most sensitive method for the immunohistological evaluation of TDP-43 pathology. However, to further dissect the potential role of phosphorylation in disease pathogenesis and the biochemical properties and potential differences of aggregated TDP-43 among TDP-43 proteinopathies, more detailed insights into additional TDP-43 phosphorylation sites are essential.

Motivated by genetic findings on a p.S375G variant in *TARDBP* in an ALS patient, the consequences of serine 375 phosphorylation of TDP-43 have recently been analyzed, with in vitro studies demonstrating a potential role for S375 phosphorylation in regulating the nuclear-cytoplasmic distribution of TDP-43 [20]. To gain further insights, we generated a novel phosphorylation-specific rabbit polyclonal antibody against TDP-43 phosphorylated at serine 375 (pTDP-43^S375^), and employed it for biochemical and detailed neuropathological analysis on a cohort of 44 sporadic and genetic ALS–TDP and FTLD–TDP cases. Phosphorylation at S375 was a consistent finding in all TDP-43 proteinopathies by immunoblot analysis. However, and most excitingly, the results of pTDP-43^S375^ immunohistochemistry (IHC) distinguished FTLD–TDP with type A pathology from other types of FTLD–TDP and ALS–TDP, most likely due to conformational differences of TDP-43 aggregates; thereby providing further evidence for the existence of distinct subtype-specific TDP-43 strains.

## Materials and methods

### Generation of polyclonal pTDP-43^S375^ antibody

The rabbit polyclonal antibody against TDP-43 phosphorylated at serine 375 was produced by Biosense/Eurogentec using their custom polyclonal antibody cross-affinity purification service. Briefly, rabbits were immunized with a phosphopeptide corresponding to amino acid 368–379 of human TDP-43: ac-EPNQAFGS(p)GNNS-C-CONH2. Phospho-specific antibodies were purified using affinity chromatography with modified and unmodified peptides. The initial validation for phospho-specificity of cross-affinity purified antibodies was performed by the company by qualitative indirect enzyme-linked immunosorbent assay (ELISA) (Supplementary Fig. 1, online resource).

### Human post-mortem cases

Human post-mortem tissues were obtained from the brain banks affiliated with the University of Tübingen, the DZNE Tübingen, and the University of British Columbia. Consent for autopsy was obtained from probands or their legal representative in accordance with local institutional review boards.

A total of 44 cases were selected covering a broad spectrum of clinical and genetic forms of primary TDP-43 proteinopathies (Table 1). These included 10 cases with clinical ALS (ALS–TDP) of which five had a pathogenic *C9orf72* repeat expansion, and 34 cases with clinical FTD (± ALS) including 15 with FTLD–TDP type A (including eight with a *GRN* mutation, and two with a *C9orf72* mutation), 11 FTLD–TDP type B (including six with the *C9orf72* repeat expansion), and 8 FTLD–TDP type C cases. Control cases included Alzheimer’s disease (*n* = 3), progressive supranuclear palsy (*n* = 2), Lewy body disease (*n* = 2), and neurologically healthy controls (*n* = 1).

**Table 1:** Demographic, clinical, and genetic profile of ALS-TDP and FTLD-TDP cases

Case	NP diagnosis	MND	Dementia	Mutation	Sex	Age at death (years)	Disease duration (years)
1	ALS-TDP	ALS	no	no	f	78	2
2	ALS-TDP	ALS	no	no	f	75	5
3	ALS-TDP	ALS	no	no	m	75	na
4	ALS-TDP	ALS	no	no	f	66	6
5	ALS-TDP	ALS	MCI	no	f	58	3
6	ALS-TDP	ALS	no	*C9orf72*	m	60	3
7	ALS-TDP	ALS	no	*C9orf72*	m	66	7
8	ALS-TDP	ALS	no	*C9orf72*	f	81	4
9	ALS-TDP	ALS	no	*C9orf72*	m	76	5
10	ALS-TDP	ALS	no	*C9orf72*	m	54	2
11	FTLD-TDP type A	no	Dementia	no	f	64	3
12	FTLD-TDP type A	no	AD	no	m	90	22
13	FTLD-TDP type A	Weakness	FTD	no	m	69	9
14	FTLD-TDP type A	no	Dementia	no	f	91	na
15	FTLD-TDP type A	no	AD	no	m	80	10
16	FTLD-TDP type A	no	FTD	*GRN*	m	66	6
17	FTLD-TDP type A	no	FTD	*GRN*	f	60	8
18	FTLD-TDP type A	Dysphagia	Dementia	*GRN*	m	72	3
19	FTLD-TDP type A	no	FTD	*GRN*	f	55	7
20	FTLD-TDP type A	no	Dementia	*GRN*	m	60	5
21	FTLD-TDP type A	no	FTD	*GRN*	f	59	9
22	FTLD-TDP type A	no	FTD	*GRN*	m	69	6
23	FTLD-TDP type A	no	FTD	*GRN*	m	64	5
24	FTLD-TDP type A	no	FTD	*C9orf72*	f	75	11
25	FTLD-TDP type A	no	FTD	*C9orf72*	m	75	10
26	FTLD-TDP type B	ALS	FTD	no	f	64	6
27	FTLD-TDP type B	no	FTD	no	m	58	5
28	FTLD-TDP type B	ALS	FTD	no	m	51	2
29	FTLD-TDP type B	ALS	Dementia	no	m	70	1
30	FTLD-TDP type B	ALS	FTD	no	f	74	3
31	FTLD-TDP type B	ALS	FTD	*C9orf72*	m	61	4
32	FTLD-TDP type B	no	FTD	*C9orf72*	m	56	7
33	FTLD-TDP type B	no	FTD	*C9orf72*	f	52	4
34	FTLD-TDP type B	no	DLB,	*C9orf72*	m	62	12
35	FTLD-TDP type B	no	FTD	*C9orf72*	m	41	6
36	FTLD-TDP type B	ALS	FTD	*C9orf72*	m	63	2
37	FTLD-TDP type C	no	FTD	no	f	70	15
38	FTLD-TDP type C	no	FTD	no	f	72	11
39	FTLD-TDP type C	no	FTD	no	f	66	9
40	FTLD-TDP type C	no	FTD	no	m	67	10
41	FTLD-TDP type C	no	FTD	no	m	69	14
42	FTLD-TDP type C	no	FTD	no	m	71	14
43	FTLD-TDP type C	no	Dementia	no	m	73	8
44	FTLD-TDP type C	no	Dementia	no	m	70	28

*AD* Alzheimer’s disease, *ALS* amyotrophic lateral sclerosis, *C9 C9orf72* repeat expansion, *f* female, *FTD* frontotemporal dementia, *GRN* granulin mutation, *m* male, *MCI* mild cognitive impairment, *MND* motor neuron disease, *na* not available

### Immunohistochemistry and immunofluorescence

IHC was performed on 2–5 μm-thick sections of formalin fixed, paraffin-embedded (FFPE) tissue using the Ventana BenchMark XT automated staining system with the optiVIEW DAB detection kit (Roche). IHC for pTDP-43^S375^ was first established on two selected cases and regions with robust TDP-43 pathology (spinal cord of ALS case, hippocampus of FTLD–TDP type B case) by testing different dilutions of the antiserum and antigen retrievals (boiling in EDTA or citrate-based buffers). For comparison, adjacent sections were stained with a phosphorylation-independent rabbit polyclonal TDP-43 antibody (ProteintechGroup).

From all ALS–TDP and FTLD–TDP cases selected for the study, sections of different CNS regions were then stained with the established pTDP-43^S375^ IHC protocol (standard IHC) with antiserum dilution 1:400, and boiling in CC1 buffer (EDTA-based buffer, Roche) for 32 min as pretreatment. Adjacent sections were stained with a rat monoclonal pTDP-43^S409/410^ antibody (clone 1D3) [19]. The anatomical regions for ALS–TDP cases included precentral gyrus, lower motor neuron (LMN) regions (hypoglossal nucleus or ventral horn of spinal cord), hippocampus, and basal ganglia. For FTLD–TDP cases, the hippocampus, temporal cortex, frontal cortex, basal ganglia, midbrain, and LMN regions were evaluated.

To investigate for conformation-depended binding of pTDP-43^S375^ antiserum, adjacent sections of selected cases were treated with 98% formic acid for 5 min before standard IHC.

Double-label immunofluorescence was performed on select sections and cases using the pTDP-43^S409/410^ antibody and the pTDP-43^S375^ antiserum. To investigate possible cross-reactivity of pTDP-43^S375^ antiserum with a subset of neurofibrillary tangles, double-label immunofluorescence was performed on AD cases using the pTDP-43^S375^ antiserum and a mouse monoclonal anti-pS202/205 tau antibody (clone AT8, ThermoFisher Scientific). Secondary antibodies were Alexa Fluor 488 conjugated anti-rabbit and Alexa Fluor 594 conjugated anti-mouse and anti-rat (Invitrogen; 1:500). 4′-6-diamidino-2-phenylindol (DAPI) was used for nuclear counterstaining.

### Semi-quantitative grading of pathology

The anatomical regions analyzed were precentral gyrus and LMN regions for ALS–TDP cases and hippocampus, temporal cortex, and LMN regions for FTLD–TDP cases. The different inclusion types evaluated for pTDP-43^S409/410^ and pTDP-43^S375^ IHC included neuronal cytoplasmic inclusions (NCI), neuronal intranuclear inclusions (NII), dystrophic neurites (DN), combined threads and dot-like profiles (ThD), white matter threads, and glial cytoplasmic inclusions (GCI). Each type of pathology was graded semi-quantitatively: 0, absent; 1, mild/occasional (easy to find but not present in every medium power field); 2, moderate (at least a few in most fields); 3, severe/abundant (many in virtually every field). In sections of hypoglossal nucleus and spinal cord, a score of 0.5 was assigned when a single LMN NCI was identified in an entire tissue section. In addition, a cumulative grade was assigned for the total amount of TDP-43-immunoreactive (-ir) pathology in each anatomical region for the respective antibodies. Grading of the pathology demonstrated by pTDP-43^S409/410^ and pTDP-43^S375^ IHC was performed blinded to the clinical and genetic information and the assigned FTLD–TDP subtype. Grading of the pathology demonstrated by pTDP-43^S375^ IHC was performed blinded to the clinical and genetic information and the assigned FTLD–TDP subtype based on pTDP-43^S409/410^ stainings.

### Sequential protein extraction

The sequential extraction of proteins with buffers of increasing stringency from fresh-frozen post-mortem frontal cortex from FTLD–TDP cases and controls was performed as described previously [17]. Briefly, gray matter was extracted at 5 ml/g (v/w) with low-salt (LS) buffer (10 mM Tris, pH 7.5, 2 mM EDTA, 1 mM dithiothreitol (DTT), 10% sucrose, and a cocktail of protease inhibitors), high-salt (HS) buffer (50 mM Tris, pH 7.5, 0.5 M NaCl, 2 mM EDTA, 1 mM DTT, 10% sucrose) with 1% Triton X-100, myelin flotation buffer (HS buffer containing 30% sucrose), and Sarkosyl (SARK) buffer (HS buffer + 1% N-lauroyl-sarcosine). The detergent-insoluble material was finally extracted in 0.25 ml/g of urea buffer (7 M urea, 2 M thiourea, 4% 3-[(3-cholamidopropyl)dimethylammonio]-1-propanesulfonate, 30 mM Tris, pH 8.5).

### Recombinant TDP-43 and in vitro phosphorylation

Human TDP-43 cDNA was subcloned into pRSET expression vector and transformed into *Escherichia coli* BL21 Star DE3 (Invitrogen). The expressed N-terminally His-tagged TDP-43 was purified under denaturing conditions using nickel beads (Qiagen) and dialyzed against NMR buffer (20 mM Na_2_HPO_4_, 20 mM NaH_2_PO_4_, 150 mM NaCl, pH 7.5). For in vitro phosphorylation, 2 µg of recombinant TDP-43 were incubated with 6 U Casein Kinase 1 δ, active (CK1; Millipore) at 30 °C for 40 min.

### Immunoblot analysis

Proteins were separated by SDS–polyacrylamide gel electrophoresis (SDS-PAGE). Immunoblot analysis was performed using fluorescence detection and the Odyssey® CLx Imaging System (LI-COR Biosciences). Proteins were transferred to nitrocellulose membranes and blocked with Odyssey blocking buffer (LI-COR Biosciences). Antibodies were detected with CF680 or CF770 conjugated anti-rat, anti-rabbit, and anti-mouse IgG (Biotium). Primary antibodies for immunoblot analysis included the rabbit pTDP-43^S375^ antiserum, clone 1D3 anti-pTDP-43^S409/410^, mouse anti-panTDP-43 (clone 6H6, own-production), and rabbit anti-panTDP-43 (#10782–2-AP, ProteinTech Group).

### Statistical analysis

Statistical analysis was performed with the GraphPadPrism software (version 7.01 for Windows). Wilcoxon test was used to assess for differences of pathology grades between pTDP^S375^ and pTDP^S409/410^ IHC. Significance level was set at *p* < 0.05.

## Results

### Basic characterization of pTDP-43^S375^ specific polyclonal antiserum

**Fig. 1:**
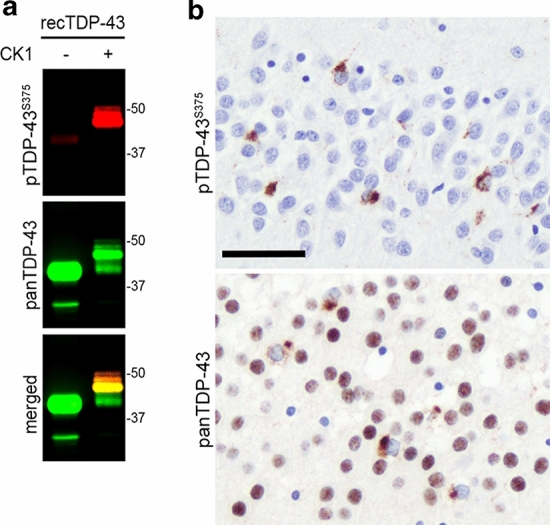
Characterization of novel pTDP-43^S375^ antiserum.** a** Immunoblot analysis of recombinant human TDP-43 before (−) or after ( +) in vitro phosphorylation by casein kinase 1 (CK1) with phosphorylation-independent TDP-43 antibody (panTDP-43, clone 6H6) and phosphorylation-specific pTDP-43^S375^ antiserum. Note that pTDP-43^S375^ antiserum only detects in vitro phosphorylated recombinant TDP-43. **b** Immunohistochemistry (IHC) for pTDP-43^S375^ labels neuronal cytoplasmic inclusions in the dentate gyrus of hippocampus from an FTLD–TDP type B case. Note that no physiological nuclear staining is observed in contrast to IHC with a phosphorylation-independent TDP-43 antibody (panTDP-43) which shows robust nuclear staining of non-inclusion bearing neurons. Scale bar: 50 µm

Specificity of affinity purified pTDP-43^S375^ antiserum for phosphorylated TDP-43 was validated by immunoblot analysis of recombinant TDP-43 before and after in vitro phosphorylation with CK1, a kinase known to phosphorylate S375 residue [8]. While untreated recombinant TDP-43 was not detected by pTDP-43^S375^ antiserum, strong labeling was seen for the in vitro phosphorylated recombinant TDP-43 (Fig. 1a). Next, the suitability of the pTDP-43^S375^ antiserum for the immunohistochemical detection of TDP-43 in human post-mortem tissue was tested by screening of selected cases with robust TDP-43 pathology and control cases. The pTDP-43^S375^ antiserum strongly labeled NCI in spinal cord LMN of an ALS–TDP case (not shown) and the dentate gyrus of an FTLD–TDP type B case (Fig. 1b). In contrast to a phosphorylation-independent TDP-43 antibody (panTDP-43), no physiological nuclear staining was detectable for pTDP-43^S375^ suggesting that S375 is not phosphorylated under physiological conditions (Fig. 1b). With the exception of nonspecific labeling of granulovacuolar degeneration and weak immunoreactivity of occasional neurofibrillary tangles in AD and PSP cases, no pTDP-43^S375^ immunoreactivity was observed in neurologically healthy controls and in Lewy body diseases (Supplementary Fig. 2, online resource). Thus, the novel antiserum is a highly sensitive and specific tool for the further investigation of the S375 phosphorylation status in TDP-43 proteinopathies.

### pTDP-43^S375^ immunoreactivity in ALS–TDP and FTLD–TDP

**Fig. 2:**
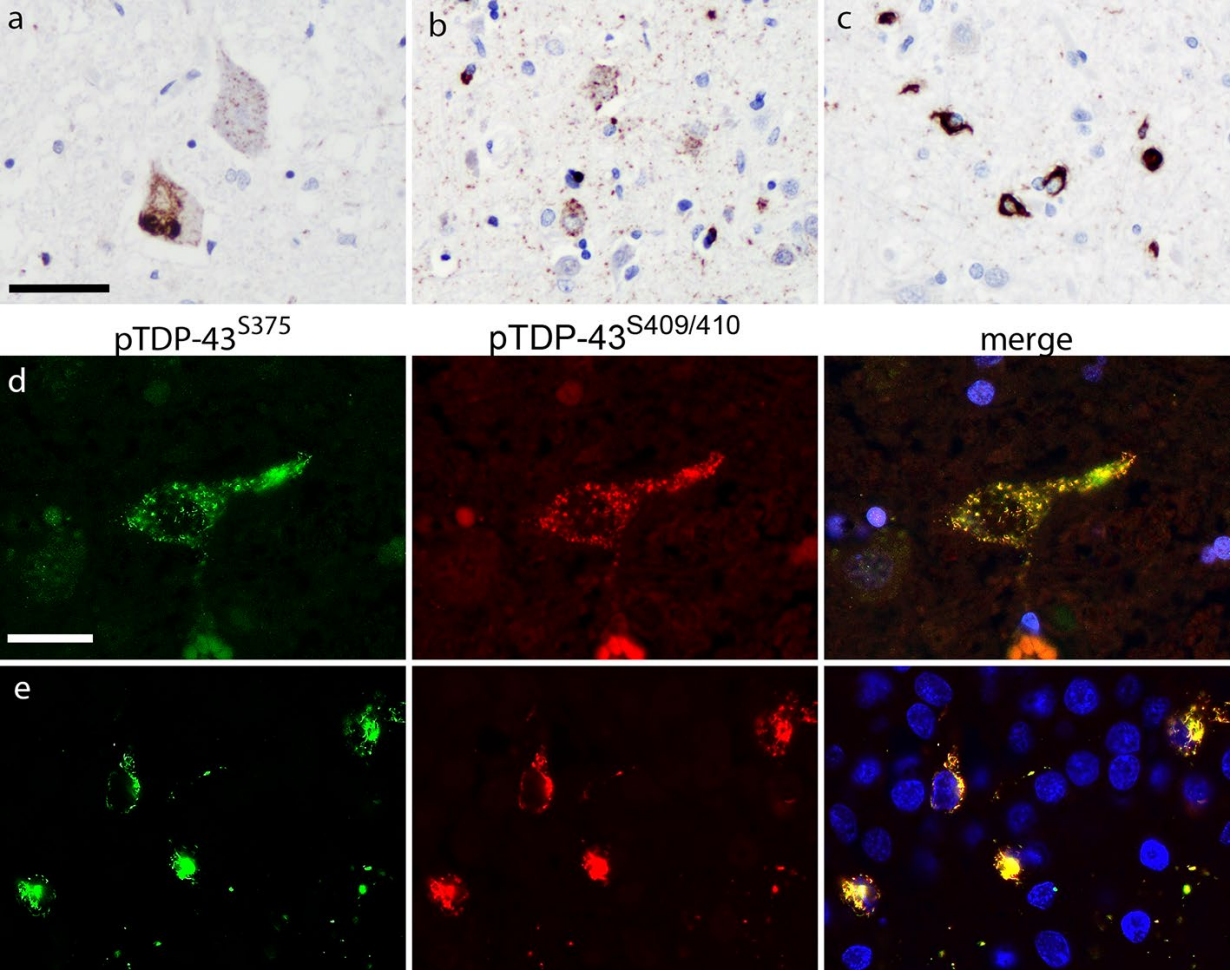
pTDP-43^S375^ immunoreactivity in ALS–TDP. pTDP-43^S375^ immunohistochemistry robustly labels the complete spectrum of pathological TDP-43 inclusions present in ALS–TDP. Specifically, diffuse and compact neuronal cytoplasmic inclusions (NCI) in the spinal cord (**a**), predominantly diffuse NCI and abundant dot/thread like neuropil staining in the precentral gyrus (**b**) and oligodendroglial inclusions in the white matter (**c**). Double-label immunofluorescence shows the complete overlap of immunoreactivity in TDP-43 pathology for pTDP-43^S375^ (green) and pTDP-43^S409/410^ (red) as shown for NCI in the spinal cord (**d**) and dentate gyrus (**e**). Nuclei stained with Hoechst (blue) in merged images. (**a**–**c**) ALS–TDP with *C9orf72* mutation; (**d**, **e**) sporadic ALS–TDP. Scale bar in **a** 50 µm (**a**, **b**), 25 µm (**c**); scale bar in **d** 25 µm (**d**, **e**)

**Fig. 3:**
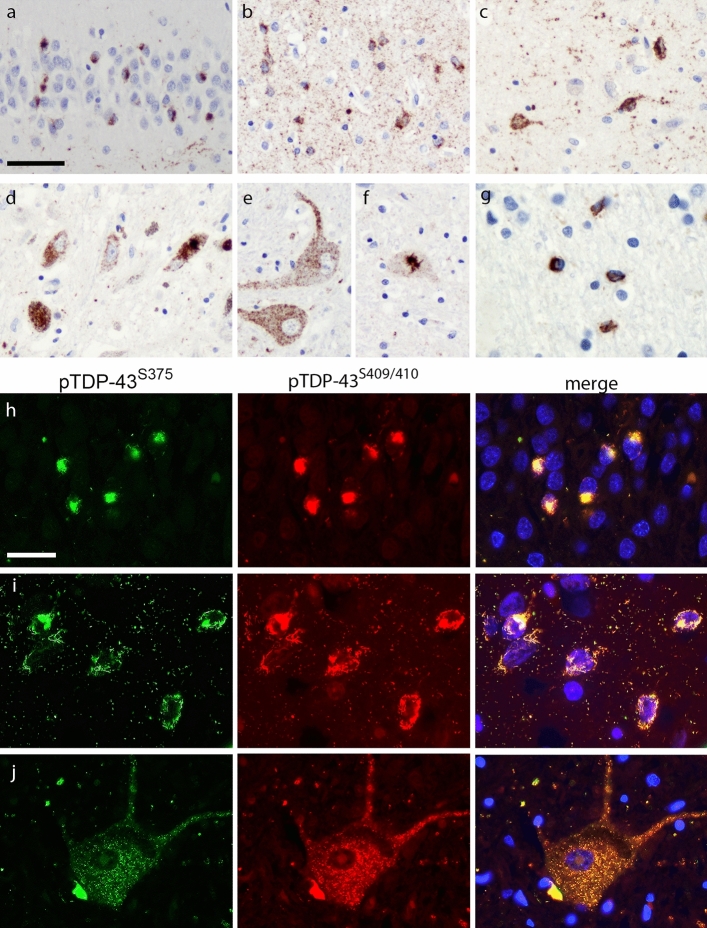
pTDP-43^S375^ immunoreactivity in FTLD–TDP type B. pTDP-43^S375^ immunohistochemistry labels all characteristic types of inclusions in cortical and subcortical regions of FTLD–TDP type B cases such as neuronal cytoplasmic inclusions (NCI) in the dentate gyrus (**a**), NCI and thread/dot pathology in the temporal cortex (**b**) and striatum (**c**), NCI in the substantia nigra (**d**), diffuse and compact NCI in the lower motor neurons (**e**, **f**), as well as oligodendroglial inclusions in the white matter (**g**). Double-label immunofluorescence for pTDP-43^S375^ (green) and pTDP-43^S409/410^ (red) confirms robust co-localization in all TDP-43-ir inclusions shown for NCI in the dentate gyrus (**h**), NCI and thread/dot pathology in frontal cortex (**i**) and NCI in spinal cord (**j**). Nuclei stained with Hoechst (blue) in merged images. (**a**, **b, e**–**g**, **j)** Sporadic FTLD–TDP type B; (**c**, **d**, **h**, **i)** FTLD–TDP type B with *C9orf72* mutation**.** Scale bar in **a**: 50 µm (**a**, **b**, **d-f**); 25 µm (**c**, **g**). Scale bar in **h** 25 µm (**h**, **i**), 33 µm (**j**)

**Fig. 4:**
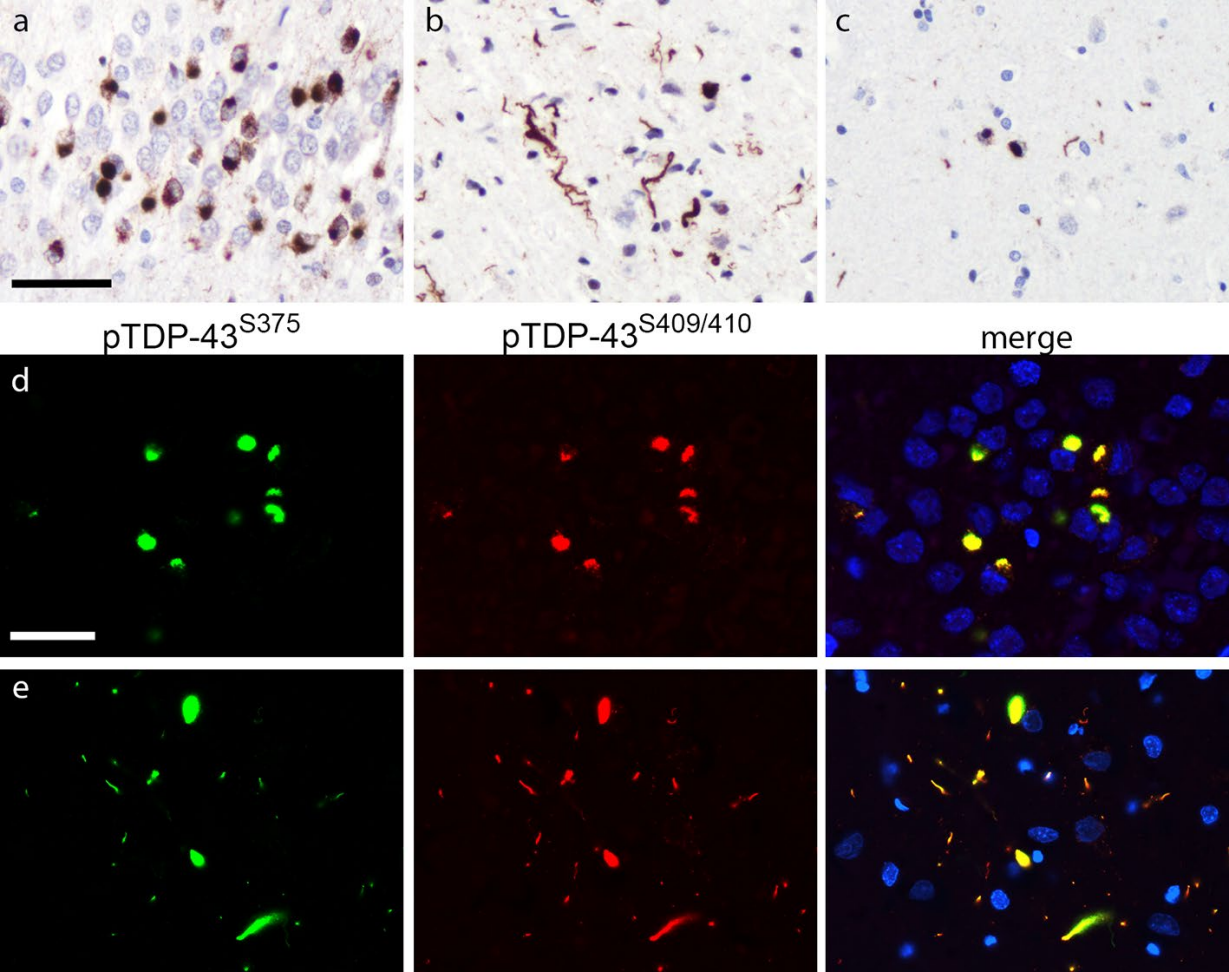
pTDP-43^S375^ immunoreactivity in FTLD–TDP type C. pTDP-43^S375^ immunohistochemistry labels all characteristic types of inclusions in FTLD–TDP type C cases including compact Pick-like neuronal cytoplasmic inclusions (NCI) in the dentate gyrus (**a**), long dystrophic neurites (DN) in the temporal cortex (**b**), and NCI and DN in the striatum (**c**). Double-label immunofluorescence shows robust co-labeling of all pTDP-43^S409/410^-immunoreactive TDP-43 inclusions (red) with antiserum against pTDP-43^S375^ (green) as shown for NCI in the dentate gyrus (**d**), and long DN in the frontal cortex (**e**). Nuclei stained with Hoechst (blue) in merged images. Scale bar in **a**: 50 µm (**a**–**c)**. Scale bar in **d**: 25 µm (**d**, **e**)

**Fig. 5:**
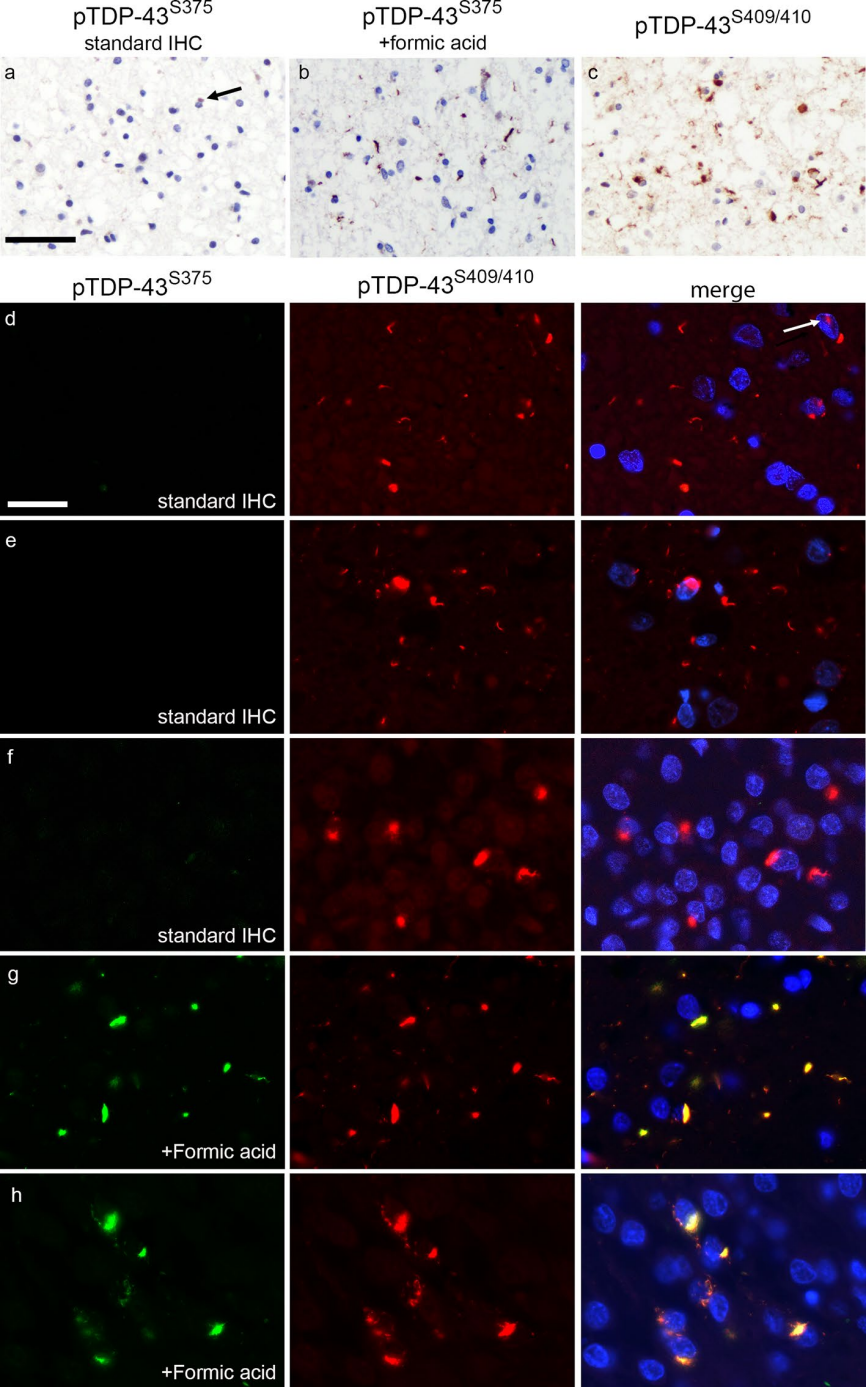
pTDP-43^S375^ immunoreactivity in FTLD–TDP type A. TDP-43 pathology of sporadic and genetic FTLD–TDP type A is almost completely negative for pTDP-4^3S375^ using the standard immunohistochemistry (IHC) protocol as illustrated for temporal cortex (**a**, arrow depicts weakly labeled neuronal cytoplasmic inclusion (NCI)). Only the following pretreatment with formic acid, numerous pTDP-43^S375^-immunoreactive NCI and dystrophic neurites (DN) become detectable (**b**) with amount of TDP-43 pathology comparable to that recognized by pTDP-43^S409/410^ IHC (**c**). Adjacent sections of the same case shown in **a**–**c**. Confirmation of findings by double-label immunofluorescence demonstrating the absence of pTDP-43^S375^ immunoreactivity (green) in pTDP-43^S409/410^-immunoreactive NCI, DN and NII (red) in the frontal cortex (**d**, arrow depicts NII), temporal cortex (**e**), and NCI in the dentate gyrus (**f**) using the standard IHC protocol. In contrast, following pretreatment with formic acid robust co-labeling is seen in inclusions for pTDP-43^S409/410^ (red) and pTDP-43^S375^ (green) shown for the temporal cortex (**g**) and dentate gyrus (**h**). Adjacent sections of the same case shown in **e** and **g**, and **f** and **h**. Sporadic FTLD–TDP type A (**d**, **f**, **h**); FTLD–TDP type A with *GRN* mutation (**a**–**c**). FTLD–TDP type A with *C9orf72* mutation (**e**, **g**). Scale bar in **a**: 50 µm (**a**–**c**). Scale bar in **c:** 25 µm (**d**–**h**)

To investigate possible differences in pTDP-43^S375^ immunoreactivity in the spectrum of TDP-43 pathology in more detail, a cohort of 44 ALS–TDP and FTLD–TDP cases, including FTLD–TDP types A-C and those with *GRN* and *C9orf72* mutations, was examined. Semi-quantitative assessment of distinct types of pTDP-43^S375^-ir inclusions in selected CNS regions was compared to the amount of TDP-43 pathology present with the pTDP^S409/410^ antibody as gold standard. As described in detail below, the whole spectrum of TDP-43-ir inclusions present in ALS–TDP, FTLD–TDP type B, and type C (i.e., diffuse and compact NCI, DN, and GCI) was consistently and strongly labeled by pTDP-43^S375^ antiserum, whereas the vast majority of inclusions in all anatomical regions examined in FTLD–TDP type A cases (i.e., diffuse and compact NCI, DN, NII, and WM treads) was not detected by pTDP-43^S375^ antiserum (Table 2; Figs. 2, 3, 4, 5).

**Table 2:** Semiquantitative assessment of pTDP-43^S375^ and pTDP-43^S409/410^ immunoreactive pathology in ALS-TDP and FTLD-TDP

	pTDP-43^S375^		pTDP-43^S409/410^	*P* value
ALS-TDP				
*Precentral gyrus (n* = *10)*				
Total	2.2 ± 0.8 [1–3]		2.3 ± 0.8 [1–3]	ns
NCI	1.8 ± 0.8 [1–3]		2.1 ± 0.8 [1–3]	ns
DN	1.7 ± 1.0 [0–3]		1.7 ± 1.0 [0–3]	ns
GCI	1.9 ± 1.0 [0–3]		1.9 ± 0.8 [1–3]	ns
*Spinal cord/medulla (n* = *10)*				
Total	2.5 ± 0.8 [1–3]		2.5 ± 0.8 [1–3]	ns
NCI	2.4 ± 0.8 [1–3]		2.4 ± 0.8 [1–3]	ns
DN	0.8 ± 0.8 [0–2]		0.8 ± 0.8 [0–2]	ns
GCI	1.5 ± 0.8 [0–3]		1.5 ± 0.8 [0–3]	ns
FTLD-TDP type A				
*Temporal (n* = *15)*				
Total	1.0 ± 0.4 [0–2]		2.6 ± 0.5 [2–3]	< 0.0001
NCI	0.5 ± 0.5 [0–1]		2.4 ± 0.8 [1–3]	< 0.0001
NII	0.3 ± 0.5 [0–1]		0.9 ± 0.5 [0–2]	0.0039
DN	1.0 ± 0.4 [0–2]		2.5 ± 0.7 [1–3]	0.0001
WM threads	0.0 [0]		1.0 ± 0.8 [0–2]	0.0020
*Dentate gyrus (n* = *13)*				
NCI	0.5 ± 0.5 [0–1]		2.1 ± 0.8 [1–3]	0.0002
*Spinal cord/medulla (n* = *13)*				
Total	0.0 [0]		0.6 ± 0.4 [0–1]	0.0039
NCI	0.0 [0]		0.2 ± 0.2 [0–0.5]	ns
DN	0.0 [0]		0.4 ± 0.5 [0–1]	ns
GCI	0.0 [0]		0.1 ± 0.3 [0–1]	ns
FTLD-TDP type B				
*Temporal (n* = *11)*				
Total	2.5 ± 0.8 [1–3]		2.5 ± 0.8 [1–3]	ns
NCI	2.2 ± 0.9 [1–3]		2.2 ± 0.9 [1–3]	ns
NII	0 [0]		0 [0]	ns
DN	2.2 ± 1.0 [0–3]		2.3 ± 1.1 [0–3]	ns
GCI	1.3 ± 1.0 [0–3]		1.3 ± 1.0 [0–3]	ns
*Dentate gyrus (n* = *11)*				
NCI	2.7 ± 0.6 [2–3]		2.7 ± 0.6 [2–3]	ns
*Spinal cord/medulla (n* = *11)*				
Total	2.5 ± 0.9 [0.5–3]		2.5 ± 0.9 [0.5–3]	ns
NCI	2.5 ± 0.9 [0.5–3]		2.5 ± 0.9 [0.5–3]	ns
DN	1.0 ± 0.8 [0–2]		1.0 ± 0.8 [0–2]	ns
GCI	1.9 ± 1.0 [0–3]		1.9 ± 1.0 [0–3]	ns
FTLD-TDP type C				
*Temporal (n* = *8)*				
Total	2.8 ± 0.7 [1–3]		2.8 ± 0.7 [1–3]	ns
NCI	1.0 ± 0.5 [0–2]		0.8 ± 0.5 [0–1]	ns
DN	2.8 ± 0.7 [1–3]		2.8 ± 0.7 [1–3]	ns
*Dentate gyrus (n* = *7)*				
NCI	3.0 ± 0.0 [3]		2.9 ± 0.4 [2–3]	ns
*Spinal cord/medulla (n* = *5)*				
Total	1.0 ± 0.7 [0–2]		1.2 ± 0.8 [0–2]	ns
NCI	0.1 ± 0.2 [0–0.5]		0.1 ± 0.2 [0–0.5]	ns
DN	1.0 ± 0.7 [0–2]		1.2 ± 0.8 [0–2]	ns
GCI	0 [0]		0 [0]	ns

*NCI* neuronal cytoplasmic inclusions, *DN* dystrophic neuritis, *GCI* glial cytoplasmic inclusions, *NII* neuronal intranuclear inclusions

Mean scores ± standard deviation [range]

*P* values: ns, not significant

#### ALS–TDP

Robust immunoreactivity with pTDP-43^S375^ antiserum was observed for all characteristic types of TDP-43 inclusion in all ALS–TDP cases examined (*n* = 10) including those with *C9orf72* mutations. Specifically, ALS–TDP showed pTDP-43^S375^-ir diffuse and compact NCI as well as DN, ThD, and GCI in the spinal cord and affected cortical regions such as the precentral gyrus, hippocampus, and basal ganglia (Fig. 2). Double-label immunofluorescence revealed robust co-localization of pTDP-43^S409/410^ and pTDP-43^S375^ immunoreactivity for all inclusions independent of the specific type (i.e., diffuse and compact NCI, GCI, DN, ThD) (Fig. 2). There were no significant differences in the amount of total TDP-43 pathology or any type of TDP-43 inclusion in the spinal cord and precentral gyrus between pTDP-43^S375^ and pTDP-43^S409/410^ (Table 2).

#### FTLD–TDP type B

IHC for pTDP-43^S375^ revealed strong and consistent labeling of neuronal and glial inclusions in all 11 cases with FTLD–TDP type B pathology including six with a *C9orf72* repeat expansion (Fig. 3). Specifically, cases showed pTDP-43^S375^-ir diffuse and compact NCI as well as DN, ThD, and GCI in affected cortical and subcortical regions such as the frontal and temporal cortex, hippocampus, striatum, midbrain, and LMNs with consistent co-localization of pTDP-43^S409/410^ and pTDP-43^S375^ observed for all inclusions by double-label IF (Fig. 3). There were no significant differences in the scores for total TDP-43 pathology or any type of TDP-43 inclusion in the spinal cord, hippocampus, and temporal cortex between pTDP-43^S375^ and pTDP-43^S409/410^ (Table 2).

#### FTLD–TDP type C

Strong pTDP-43^S375^ immunoreactivity was observed in the characteristic inclusions of type C, i.e., long DN in the neocortex and round compact NCI in the dentate gyrus and striatum in all eight FTLD–TDP type C cases analyzed (Fig. 4). The amount of TDP-43 pathology was similar to that observed for pTDP-43^S409/410^ IHC as illustrated by double-label IF showing complete co-localization for pTDP-43^S409/410^ and pTDP-43^S375^ in all inclusions and absent differences in the scores in our semiquantitative evaluation of TDP-43 inclusions in the hippocampus and temporal cortex (Table 2).

#### FTLD–TDP type A

The cohort of FTLD–TDP type A cases consisted of 15 cases including eight with a *GRN* mutation and two with a *C9orf72* mutation. All cases showed moderate-to-severe TDP-43 pathology detected by pTDP-43^S409/410^ IHC in the cortical and subcortical regions characteristic for FTLD–TDP type A with small compact NCI, DN, and NII in the frontotemporal gray matter, thread pathology in the white matter, diffuse, and compact NCI in the dentate gyrus and threads in the CA1 region of the hippocampus [13, 14]. However, with the exception of a few very weakly labeled NCI, DN, and NII in a subset of cases, the vast majority of any type of TDP-43-ir pathology in type A cases (i.e., NCI, DN, NII, WM threads) did not label for pTDP-43^S375^ using the standard IHC protocol (Fig. 5a). The absence of pTDP-43^S375^ immunoreactivity in NCI, DN, NII, and WM threads was a consistent finding in all cases and in all examined CNS regions including frontal and temporal cortex, basal ganglia, midbrain, and spinal cord as further confirmed by double label IF (Fig. 5d–f). Scores for total TDP-43 pathology and all types of inclusions analyzed (i. e., NCI, DN, NII, WM threads) in our semiquantitative evaluation of TDP-43 pathology in the hippocampus and temporal cortex were significantly lower for pTDP-43^S375^ compared to pTDP-43^S409/410^ (Table 2). Notably, there were no differences between type A cases associated with a *GRN* mutation or with a *C9orf72* mutation and there were no differences between sporadic and genetic type A cases, indicating that the lack of immunoreactivity is closely associated with the morphological subtype of TDP-43 pathology, regardless of the underlying cause.

To address the intriguing possibility that the antibody selectivity might depend on the conformational status of TDP-43 aggregates in type A inclusions in which the epitope is buried, selected sections were subjected to formic acid (FA) treatment for 5 min before standard IHC. Notably, only under these harsh denaturing conditions, TDP-43 pathology became pTDP-43^S375^ immunoreactive (Fig. 5b, g, h).

### Biochemical assessment pTDP-43^S375^ in FTLD–TDP

**Fig. 6:**
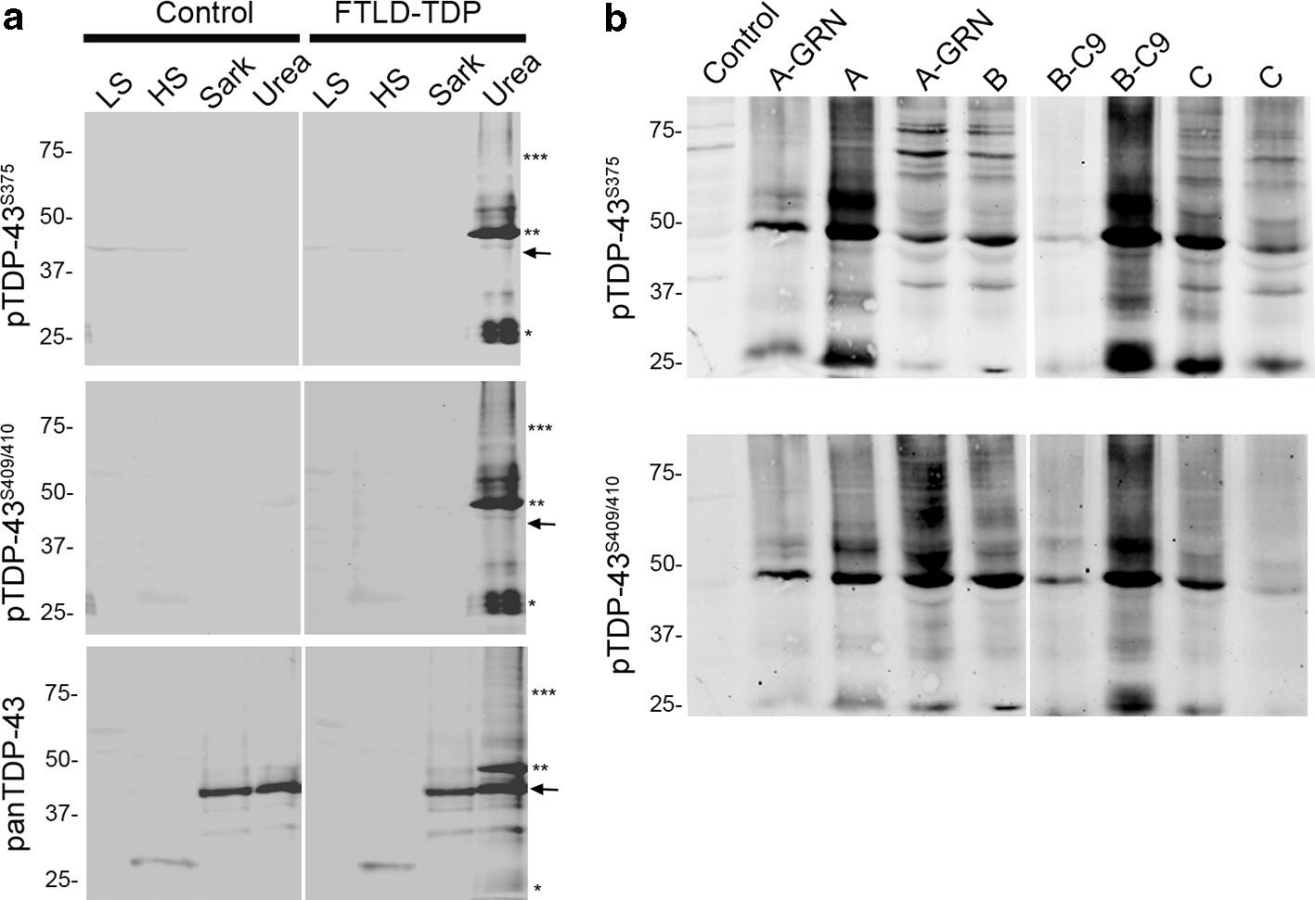
Biochemical characterization of pTDP-43^pS375^ in human post-mortem tissue. **a** Immunoblot analysis of sequentially extracted protein fractions from FTLD–TDP and control brain with phosphorylation-specific and phosphorylation-independent (panTDP) TDP-43 antibodies. With the panTDP-43 antibody, the typical pattern of pathologic TDP-43 species in the urea fraction of FTLD–TDP is seen with bands ~ 45 kDa (**), ~ 25 kDa (C-terminal fragments, *), and a high molecular smear (***) in addition to the physiological TDP-43 present as ~ 43 kDa band (arrow) in the sarkosyl and urea fraction in FTLD–TDP and control. Note that pTDP-43^S375^ antiserum labels only the pathological TDP-43 species in the urea fraction similar to results with an antibody against pTDP-43^S409/410^. **b** Immunoblot of urea fractions from FTLD–TDP type A, B, and C cases showing moderate-to-strong labeling of pathological TDP-43 species for pTDP-43^S375^ in all subtypes with comparable intensities as seen for pTDP-43^S409/410^

To gain further insights on the presence of pTDP-43^S375^ in TDP-43 proteinopathies, we performed immunoblot analysis using protein lysates sequentially extracted from frozen brain tissues of cases and controls. The disease-specific TDP-43 signature was observed with pTDP-43^S375^ antiserum in the urea fraction with specific labeling of abnormal TDP-43 species including hyperphosphorylated full-length TDP-43 (band ~ 45 kDa), C-terminal fragments (bands ~ 20-25 kDa), and a high molecular smear. No band ~ 43 kDa corresponding to physiological TDP-43 was detectable in any fraction in FTLD–TDP or in controls. The biochemical signature observed for pTDP-43^S375^ resembles that seen with other phosphorylation-specific antibodies, i.e., pTDP-43^S409/410^ (Fig. 6 a).

Notably, the presence of pTDP-43^S375^ in the urea fraction was a consistent finding in all sporadic and genetic FTLD–TDP types examined including type A (Fig. 6b), thereby excluding that the minimal immunoreactivity observed by IHC in type A cases is due to a lack of pTDP-43^S375^. Together with the results of IHC following FA pretreatment, these findings support the interpretation that FTLD–TDP type A cases are characterized by accumulation of pathological TDP-43 that has a unique conformation that renders the pTDP-43^S375^ epitope inaccessible for standard IHC.

## Discussion

Accumulation of hyperphosphorylated TDP-43 is the hallmark pathological feature of FTLD–TDP and ALS–TDP. However, most of the specific molecular modifications, their pathogenic relevance, and diagnostic potential remain to be identified. Here, by employing a novel phosphorylation-specific TDP-43 antibody, we demonstrate that phosphorylation of S375 is a consistent feature of pathological TDP-43 in sporadic and familial forms of FTLD–TDP and ALS–TDP. Most excitingly, the antibody revealed striking differences among cases by IHC with consistent binding to TDP-43 inclusions in ALS–TDP and FTLD–TDP type B and C cases but not to the TDP-43 inclusions in FTLD–TDP type A cases. These data are suggestive of a conformation-selective binding of pTDP-43^S375^ antiserum, thereby supporting the concept of distinct TDP-43 strains as the molecular basis for the phenotypic diversity in TDP-43 proteinopathies.

The specificity of the novel pTDP-43^S375^ antiserum for phosphorylated TDP-43 species was validated by ELISA and by biochemical analysis of recombinant TDP-43 with/without in vitro phosphorylation by CK1, an assay which has been previously reported by mass spectrometry to in vitro phosphorylate TDP-43 at S375 [8]. Phosphorylation of TDP-43 at S375 has been reported so far in two ALS–TDP cases by mass spectrometry of sarcosyl-insoluble protein fractions isolated from brains [9]. Our biochemical analysis of human post-mortem brain tissue probed with the novel antiserum verify and expand this finding by demonstrating that phosphorylation of S375 is a consistent feature of pathological TDP-43 species in FTLD–TDP types A–C and ALS–TDP, thereby adding pS375 to the list of validated and confirmed abnormal PTM in TDP-43 proteinopathies. The observed banding pattern in immunoblot analysis of sequential protein extracts isolated from diseased brains with highly specific labeling of pathological full-length TDP-43, C-terminal fragments, and high molecular smear in the urea fraction of FTLD–TDP and ALS–TDP without labeling of the physiological TDP-43 at ~ 43 kDa was comparable to the profile seen with antibodies specific for TDP-43 phosphorylated at other serine residues (S379, 403, 404, 409, 410) [4, 6, 19] and suggests that S375 phosphorylation is an abnormal event.

Phosphorylation-specific antibodies with those against pS409/410 as the most widely used are the most sensitive and specific markers for the detection of the complete spectrum of TDP-43 pathology by IHC [4, 19, 28]. When we employed the novel pTDP-43^S375^ antibody for a detailed immunohistochemical analysis of TDP-43 pathology in a cohort of ALS–TDP and FTLD–TDP cases, covering the most common pathological and genetic subtypes, striking differences were observed among subgroups. In sporadic and genetic ALS–TDP and FTLD–TDP type B, including cases with *C9orf72* mutations, as well as in type C cases, TDP-43-ir pathology was consistently and strongly labeled by pTDP-43^S375^ antiserum including all distinct types of inclusions (e.g., diffuse and compact NCI, short and long DN, GCI). In contrast, almost all TDP-43-ir inclusions (i.e., NCI, DN, NII, and WM threads) in sporadic and genetic FTLD–TDP type A cases, including cases with *GRN* and *C9orf72* mutations, were completely negative. Notably, the immunoreactivity profile of TDP-43-ir pathology for pTDP-43^S375^ was closely associated and determined by the morphological pattern of TDP-43, regardless of the underlying cause.

Several possibilities can be excluded as explanation for the discordant staining properties of TDP-43-ir inclusions between FTLD–TDP type A and others. First, we can exclude that the lack of pTDP-43^S375^ immunoreactivity by IHC in FTLD–TDP type A is simply a result of absent phosphorylation of TDP-43 at S375 in type A since our biochemical studies demonstrated the consistent presence of pTDP-43^S375^ in all cases including FTLD–TDP type A. Second, it is known that various factors such as fixation time, fixative, and post-mortem delay might influence the detectability of antigens in tissues. However, this possibility also seems highly unlikely, since these parameters did not differ between FTLD–TDP type A, and in other cases, no correlation was observed between the intensity of immunoreactivity and these factors in ALS–TDP and FTLD–TDP type B/C, and moreover, the same distinction of type A pathology from TDP-43 pathology in other subgroups was seen in tissues sampled at two different brain banks.

In fact, our data rather imply that TDP-43 species in type A inclusions have a different structural assembly compared to aggregates in other FTLD–TDP types and ALS–TDP in which the epitope recognized by the pTDP-43^S375^ antibody is buried and not accessible. The interpretation that the novel antibody recognizes TDP-43 in a conformational-dependent and type-specific manner is corroborated by the fact that FTLD–TDP type A inclusions became immunoreactive when FFPE sections were subjected to very harsh denaturing conditions by formic acid treatment for 5 min before IHC. Thus, our data provide strong evidence in support of the popular idea of biochemically distinct TDP-43 species (i.e., strains) as the molecular basis of disease heterogeneity among TDP-43 proteinopathies. In this context, it is noteworthy that the postulated distinct biochemical property/conformation of aggregated TDP-43 recognized by our novel antibody is conserved throughout different affected CNS regions, cell types (i.e., neurons and oligodendrocytes), and inclusion types (i.e., NCI, NII, and DN) in a given subtype, indicating a common mechanism for the formation of all inclusions in an individual, regardless of the cell type and intracellular compartment, consistent with a prion-like propagation of TDP-43 aggregation in a self-templating manner.

Evidence for biochemical heterogeneity of aggregated TDP-43 has been reported previously by description of different patterns of C-terminal fragments, protease-resistant cores, and distinct biophysical properties of aggregated TDP-43 among FTLD–TDP subtypes [10, 11, 29]. While it remains to be seen, whether these biochemical differences, indeed, represent strains with specific cell tropism and toxicity, there are several reports emerging that support this view by description of distinct seeding activities and toxicity of TDP-43 aggregates in vitro and in vivo [11, 22, 23]. Moreover, the mechanism and triggers for the formation of distinct conformational states of TDP-43 remain to be established. The fact that we found the immunoreactivity profile of TDP-43 inclusions for pTDP-43^S375^ to be determined by the morphological pattern of TDP-43, regardless of the underlying gene defect in genetic cases (i.e., *GRN, C9orf72*), suggests an involvement of additional factors. Our data are in line with the previous findings by Laferriere et al. in which biochemical/biophysical properties of TDP-43 aggregates were also reported to be better predicted by the pathological subtype than by the genetic cause [11].

The aim of the current study was to investigate pathological subtype-specific differences; therefore, we focused on the most common primary TDP-43 proteinopathies. However, other rarer FTLD–TDP subtypes (e.g., type D associated with *VCP* mutations and type E) and other genetically based ALS/FTLD–TDP cases (e.g., associated with mutations in *TARDBP*, *UBQLN2*, *OPTN*, and *TBK1*) exist and it will be interesting to learn in the future how they will be classified based on pTDP-43^S375^ immunoreactivity [12, 18]. Moreover, it is expected that the antibody will be a powerful tool to further dissect the biochemical basis of occasional FTLD–TDP cases with difficult to classify and/or mixed patterns of pathology that seem to be particularly common in *C9orf72* mutation carriers [13, 14, 21, 26, 27] and to identify potential molecular overlaps of TDP-43 aggregates in FTLD–TDP with those in secondary TDP-43 proteinopathies, e.g., cases with anatomically restricted TDP-43 pathology in association with other common neurodegenerative diseases and aging [7].

Another important question to be addressed in the future remains the potential role of S375 phosphorylation for physiological TDP-43 functions and in disease pathogenesis. Our findings with absent labeling for the physiological normal TDP-43 by immunoblot analysis of cultured cells (Supplementary Fig. 3, online resource) and human brain homogenates and lack of physiological nuclear staining in non-inclusion bearing cells by IHC suggest that phosphorylation of S375 is an abnormal event. However, we cannot rule out that phosphorylation at S375 might occur physiologically to a minor extent or under specific stress conditions. Cell culture studies with phosphomimetic TDP-43 mutations suggest that phosphorylation of S375 might modulate nuclear-cytoplasmic shuttling leading to a higher tendency to accumulate in the cytoplasm particularly under cellular stress conditions [20]. A potential role in aggregation is supported by the localization of S375 in the C-terminal low-complexity domain of TDP-43, a region known to be involved in TDP-43 phase separation, intermolecular interactions, and aggregation. Notably, residue 375 is included in one of the six reported steric-zipper structures of TDP-43 shown to be important for TDP-43 aggregation with postulated segmental polymorphs [3].

In summary, we describe phosphorylation of TDP-43 at S375 as a consistent feature in sporadic and genetic ALS-TPD and FTLD–TDP and demonstrate that standard staining protocols using this novel antibody allows the distinction of TDP-43 pathology of FTLD–TDP type A from other FTLD–TDP types and ALS–TDP, most likely in a conformation-dependent manner, compatible with the concept of alternate TDP-43 conformers as the biochemical basis for phenotypic diversity. The newly generated pTDP-43^S375^ antibody will not only be a valuable tool to further address the role of pS375 in disease pathogenesis, but also as an aid in subtyping of FTLD–TDP, particularly for difficult to classify cases. However, we recognize that the latter requires the validation of our discriminatory IHC approach by other research groups in their FTLD–TDP cohorts.

## Electronic supplementary material

Below is the link to the electronic supplementary material.

Supplementary file 1 (PDF 605 kb)
