# Supplementary material for: Antibody against TDP-43 phosphorylated at serine 375 suggests conformational differences of TDP-43 aggregates among FTLD–TDP subtypes
Source: Acta Neuropathol. 2020 Aug 10;140(5):645–58. doi: 10.1007/s00401-020-02207-w (PMC7547034; doi:10.1007/s00401-020-02207-w)
Supplement: Supplementary file 1 — (PDF 605 kb) [file 401_2020_2207_MOESM1_ESM.pdf]

Antibody against TDP-43 phosphorylated at serine 375 suggests conformational differences of TDP-43 aggregates among FTLD-TDP subtypes

Manuela Neumann[1,2], Petra Frick[1], Francesca Paron[3], Jonas Kosten[1], Emanuele Buratti[3], Ian R. Mackenzie[4]

Supplementary online material:

Supplementary figure 1: Qualitative enzyme-linked immunosorbent assay (ELISA) for pTDP-43^{S375} antibody
Supplementary figure 2: pTDP-43^{S375} immunohistochemistry in controls
Supplementary figure 3: Immunoblot of HEK293 cell lysates with pTDP-43S375 antibody

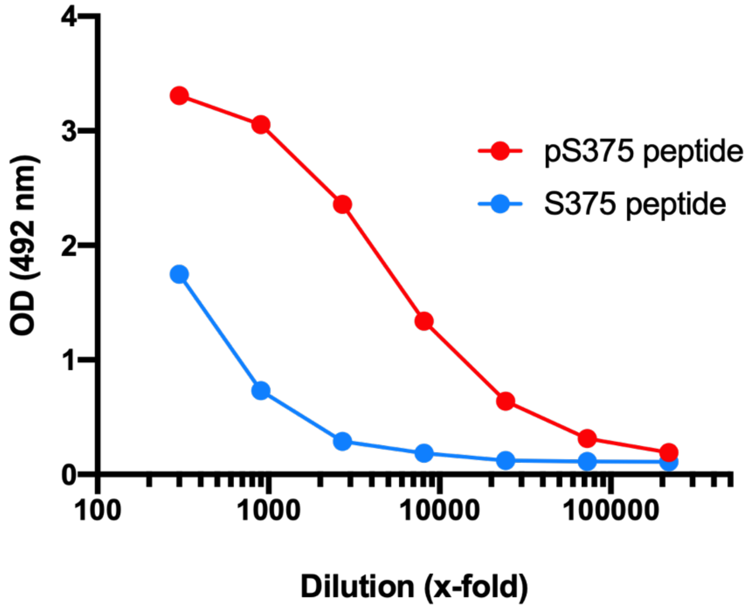

Supplementary figure 1: Qualitative enzyme-linked immunosorbent assay (ELISA) for pTDP-43^{S375} antibody

Indirect ELISA performed with serial dilutions of cross-affinity purified antibody pTDP-43^{S375} against peptides (5-15 µg/well) corresponding to amino acids 368-379 of human TDP-43 with serine 375 phosphorylated (pS375 peptide) or non-phosphorylated (S375 peptide). Data generated by Biosense/Eurogentec as part of their custom polyclonal antibody cross-affinity purification service.

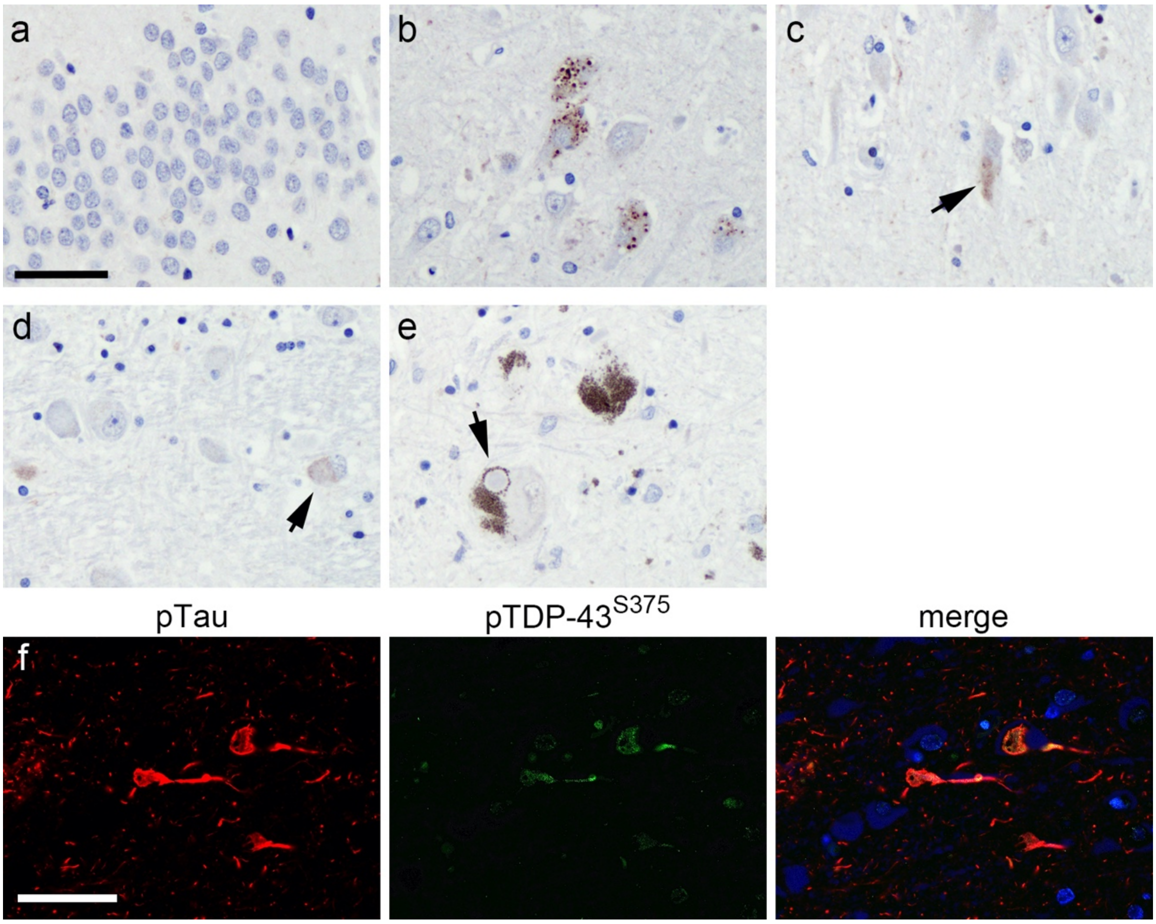

Supplementary figure 2: pTDP-43^{S375} immunohistochemistry in controls

No labeling is seen with pTDP-43^{S375} antiserum in neurologically healthy controls (dentate gyrus) (**a**). In Alzheimer's disease moderate to strong immunoreactivity is present in granulovacular degeneration in hippocampal neurons (**b**) and weak immunoreactivity in a subset of neurofibrillary tangles (arrow in **c**). Weak immunoreactivity is also seen in subset of neurofibrillary tangles in PSP case (arrow in **d**). Lewy bodies in Parkinson's disease are not stained (arrow in **e**). Weak co-labeling of AT8-positive (red) neurofibrillary tangles with pTDP-43^{S375} (green) demonstrated by double-label immunofluorescence in Alzheimer's disease (**f**).

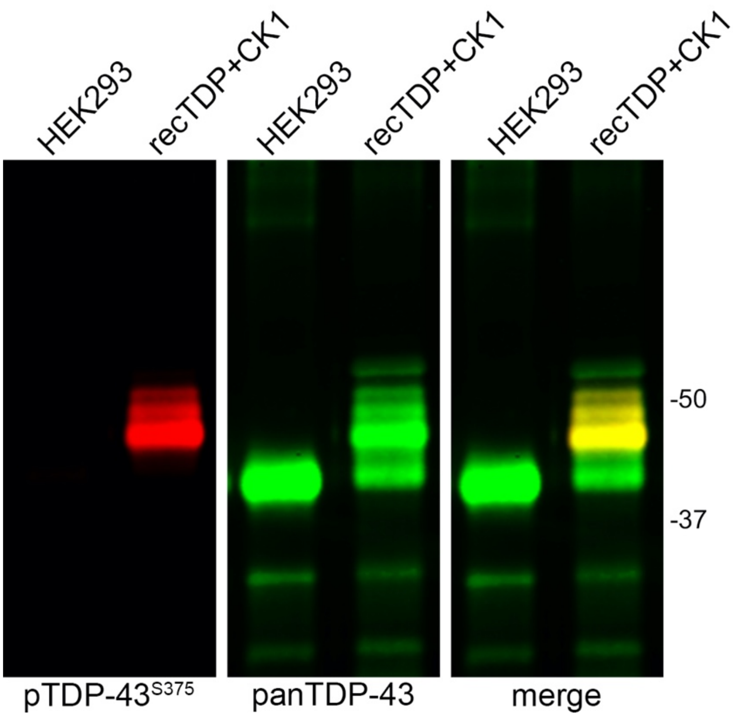

Supplementary figure 3: Immunoblot of HEK293 cell lysates with pTDP-43^{S375} antibody

Immunoblot of RIPA lysates from HEK293 cells and recombinant TDP-43 *in vitro* phosphorylated by casein kinase 1 (CK1) used as positive control probed with phosphorylation-independent TDP-43 antibody (panTDP-43, clone 6H6) and phosphorylation specific pTDP-43^{S375} antiserum. Note that no signal is detectable for pTDP-43^{S375} antiserum in cell culture lysate.
